# FMLNCSIM: fuzzy measure-based lncRNA functional similarity calculation model

**DOI:** 10.18632/oncotarget.10008

**Published:** 2016-06-14

**Authors:** Xing Chen, Yu-An Huang, Xue-Song Wang, Zhu-Hong You, Keith C.C. Chan

**Affiliations:** ^1^ School of Information and Electrical Engineering, China University of Mining and Technology, Xuzhou, China; ^2^ Department of Computing, Hong Kong Polytechnic University, Hong Kong; ^3^ School of Computer Science and Technology, China University of Mining and Technology, Xuzhou, China

**Keywords:** lncRNAs, functional similarity, disease, fuzzy measure, directed acyclic graph

## Abstract

Accumulating experimental studies have indicated the influence of lncRNAs on various critical biological processes as well as disease development and progression. Calculating lncRNA functional similarity is of high value in inferring lncRNA functions and identifying potential lncRNA-disease associations. However, little effort has been attempt to measure the functional similarity among lncRNAs on a large scale. In this study, we developed a Fuzzy Measure-based LNCRNA functional SIMilarity calculation model (FMLNCSIM) based on the assumption that functionally similar lncRNAs tend to be associated with similar diseases. The performance improvement of FMLNCSIM mainly comes from the combination of information content and the concept of fuzzy measure, which was applied to the directed acyclic graphs of disease MeSH descriptors. To evaluate the effectiveness of FMLNCSIM, we further combined it with the previously proposed model of Laplacian Regularized Least Squares for lncRNA-Disease Association (LRLSLDA). As a result, the integrated model, LRLSLDA-FMLNCSIM, achieve good performance in the frameworks of global LOOCV (AUCs of 0.8266 and 0.9338 based on LncRNADisease and MNDR database) and 5-fold cross validation (average AUCs of 0.7979 and 0.9237 based on LncRNADisease and MNDR database), which significantly improve the performance of previous classical models. It is anticipated that FMLNCSIM could be used for searching functionally similar lncRNAs and inferring lncRNA functions in the future researches.

## INTRODUCTION

In recent years, the observation from the Next Generation Sequencing (NGS) project indicates that the number of non-coding sequences accounts for a large portion (more than 98%) of the complete human genome. A great number of non-coding RNAs (ncRNAs) are discovered which do not encode proteins, especially long noncoding RNAs (lncRNAs). LncRNA is the heterogeneous ncRNAs which consist of more than 200 nucleotides. According to the relative positions to the coding genes, there are five subgroups of lncRNAs (i.e. sense, antisense, bidirectional, intronic, and intergenic) [1–3]. As a traditional viewpoint from central dogma of molecular biology, the genetic information is mainly stored in the protein-coding genes. The special characters of lncRNAs, low expression level and high tissue-specific pattern, once leaded to a misconception that lncRNAs are purely “transcriptional noise” [4–6]. However, increasing evidences from biological experiments have shown that lncRNAs carry out various crucial functions, which clearly contradict to the traditional viewpoint. LncRNAs cover a wide range of functions of modulating gene expression at the epigenetic, transcriptional, and post-transcriptional levels [2]. Specifically, lncRNAs get involved in diverse biological processes, such as chromatin modification, cell differentiation and proliferation, RNA progressing, and cellular apoptosis [7–14]. For example, HOTAIR was verified as scaffold to bind histone modifiers, PRC2, and the LSD1 complex, carrying out functions of histone modifications control and gene expression regulation [15]. Xist also proved to be a spliced and polyadenylated lncRNA which binds and recruits PRC2 to initiate X chromosome inactivation [16]. UCA1 is discovered to regulate the expression of several genes which are involved in tumorigenesis and embryonic development [17].

According to the new theory of competing endogenous RNA, lncRNAs interact with a wide range of RNA molecules and play a more important role in pathological conditions than previously expected [18]. Based on the existing experimental observations, lncRNAs emerge as important drivers of diverse diseases and modulate gene expression at several levels. The competing endogenous RNAs are considered to be involved in a large-scale regulatory network across the transcriptome, playing important roles in pathological conditions. Increasing evidence indicates that the lncRNA dysfunction is clearly associated with the development and progression of a wide range of diseases, such as diabetes [19, 20], HIV [21], breast cancer [22, 23], lung cancer [24, 25], colon cancer [26], prostate cancer [20], leukemia [27], and ovarian cancer [28]. Some lncRNAs are considered as biomarkers for specific diseases. For example, M41 was verified as a biomarker candidate for the prognosis of ER-associated breast cancers [29]. It was identified to be associated with preclinical cancer phenotype, tamoxifen resistance promotion, and poor outcomes in clinical samples. Except for M41, ANRIL was recently regarded as a potential prognostic biomarker in gastric cancer [30]. It recruits and binds to PRC2 and generally upregulates in human gastric cancer tissues. SNHG18 was also identified as a predictive biomarker for bladder cancer. The relevant study shows that the knockdown of SNHG18 can lead to decreased expression of several luminal PPARγ target genes including uroplakins and fibroblast growth factor receptor-3 (FGFR3), and further boost the development of muscle-invasive bladder cancer [31]. Even though the mechanisms of complex diseases are still unclear, the biological data collected from experimental discoveries is expected to shed light on the roles of lncRNAs in disease development and progression.

With the rapid development of experimental techniques and computational studies for lncRNA discovery, a large number of lncRNAs in various eukaryotic organisms have been discovered since H19 and XIST were first discovered in the early 1990s [32–34]. Many lncRNA-related biological datasets have been built and stored in some publicly available databases, such as NRED [35], NONCODE [36] and LncRNAdb [11]. However, the number of lncRNA-disease associations recorded in these databases is still limited. Even though more and more lncRNA functions have been identified by the disease-related studies, it is unrealistic to use experimental approaches to identify the functions of lncRNAs due to the high cost of time and money. In recent years, lncRNA-disease association identification and lncRNA function prediction have become hot research subjects attracting an increasing number of researchers. Based on the assumption that similar lncRNA functions are associated with the involvement in similar diseases, some computational models have been reported to calculate lncRNA functional similarity or identify lncRNA-disease associations [37–41]. These methods are mainly based on the recorded lncRNA-disease association networks. Due to the rapid computational process and the integration of various types of biological data, computational models can serve as a perfect complement for biological experiments by calculating lncRNA functional similarity or selecting the most probable candidate for further experimental validation. Therefore, computational methods for lncRNA-related studies are drawing increase attentions and expected to decrease the time and cost of experimental approaches [42–46]. In conclusion, developing computational models for calculating lncRNA functional similarity could not only boost the understanding on disease mechanism at lncRNA level, but also accelerates the process of new biomarker identification for drug discovery, disease diagnosis, treatment, prognosis, and prevention.

Currently, measuring lncRNA functional similarity is still a challenging task. The difficulties lie in the undocumented structural features and weak conservation of lncRNA structures as well as the lack of reliable network model for uncovering the relationships between lncRNAs and other molecules [47]. However, accumulating studies provide the clues that mutations in the primary structure, secondary structure, expression levels and cognate RNA-binding proteins of lncRNAs underlie their biological functions [9, 48, 49]. Therefore, based on this fact, there are some computational models have been proposed for measuring lncRNA functional similarity by utilizing diverse expression profiles of lncRNAs. For example, Bellucci *et al.* proposed a method for measuring lncRNA functions by considering their potential associated proteins which were predicted based on sequence information [50]. In addition, Chen *et al.* reported a novel measurement of integrated lncRNA functional similarity by combining lncRNA expression similarity with lncRNA Gaussian interaction profile kernel similarity [37]. In this study, the lncRNA expression similarity was defined as the Spearman correlation coefficient between the expression profiles of each lincRNA pairs. Based on this measurement for lncRNA functional similarity, the first lncRNA-disease association prediction model of LRLSLDA was proposed. For lncRNA functional measurement, Liao *et al.* proposed a model based on coding-noncoding gene co-expression network which was constructed by re-annotating probes of the Affymetrix Mouse Genome 430 2.0 Array [51]. However, this kind of expression data suffers from the strong dependence on the design of the probes. Xiao *et al.* also recently proposed a model by mapping protein-coding genes onto human PPI network based on Bayesian network [52]. In this work, lncRNA functional similarity could be measured by mining highly connected molecules in the network. In recent years, it's worth noting that the involvement of lncRNAs in a wide range of diseases could be far more prevalent than previously considered. Based on the assumption that lncRNAs which get involved in similar diseases are more likely to share the similar biological functions and vice versa, some computational models for calculating lncRNA functional similarity have been proposed. For example, Sun *et al.* proposed a calculation model for lncRNA functional similarity by adopting DOsim, an R package proposed by Wang *et al.*, to measure semantic similarity between disease directed acyclic graphs (DAGs) [53, 54]. Chen *et al.* developed the model of LFSCM for measuring lncRNA functional similarity [38]. This model was mainly based on the combination of microRNA (miRNA)-disease associations with lncRNA-miRNA interactions. Recently, Chen *et al.* further reported a computational method named LNCSIM for calculating lncRNA functional similarity by combining the experimentally confirmed lncRNA-disease associations and the information of DAGs constructed by disease Mesh descriptors [55]. The effectiveness of this kind of method mainly depends on the term-term similarity measure on the collocations derived from DAGs. In the model of LNCSIM, the traditional Jaccard similarity measure was adopted for calculating the semantic similarity between each disease pairs. With the continuous emergence of new clinical discoveries, this kind of method which uses know lncRNA-disease association can greatly benefit from the wealth of observation data. It is a current trend for network-based prediction models to consider additional biological knowledge [56–58].

In this study, we developed Fuzzy Measure-based LNCRNA functional SIMilarity calculation model (FMLNCSIM) based on the assumption that similar diseases tend to be involved with functionally similar lncRNAs and vice versa. The model of FMLNCSIM generally consists of two parts. In the first part, the terms of MeSH descriptors in a combined set describing two diseases would be considered as “information sources” which were used for calculating the similarities among diseases. Specifically, the semantic similarities of diseases were computed by combining the concepts of information content and fuzzy measure. In the second part, the functional similarity of two lncRNAs would be calculated based on the semantic similarities of their associated disease groups. To further evaluate the effectiveness of FMLNCSIM, we used the calculation results computed from FMLNCSIM to predict the lncRNA-disease associations based on the model of LRLSLDA which was proposed in the previous work. The performance of new integrated model would be directly influenced by FMLNCSIM and therefore reflected effectiveness of FMLNCSIM. We used two evaluation frameworks, namely global leave-one-out cross validation (LOOCV) and 5-fold cross validation, to evaluate the performance of FMLNCSIM. When exploring the LncRNADisease and MNDR databases by adopting the global LOOCV method, we obtained improved performance with AUCs of 0.8266 and 0.9338, respectively. By adopting 5-fold cross validation method based on LncRNADisease and MNDR databases, FMLNCSIM yielded average AUCs of 0.7979 and 0.9237, respectively. In addition, we further verified the top 10 prediction lists of acute myeloid leukemia and lung cancer by checking the updates of relevant databases and recent experimental literatures. As a result, six of them were confirmed. These reliable results demonstrate that FMLNCSIM is feasible and promising to quantify functional similarity of lncRNAs as well as to be combined with similarity-based computational models for lncRNA-disease association prediction.

## RESULTS

### Model design

**Figure 1:**
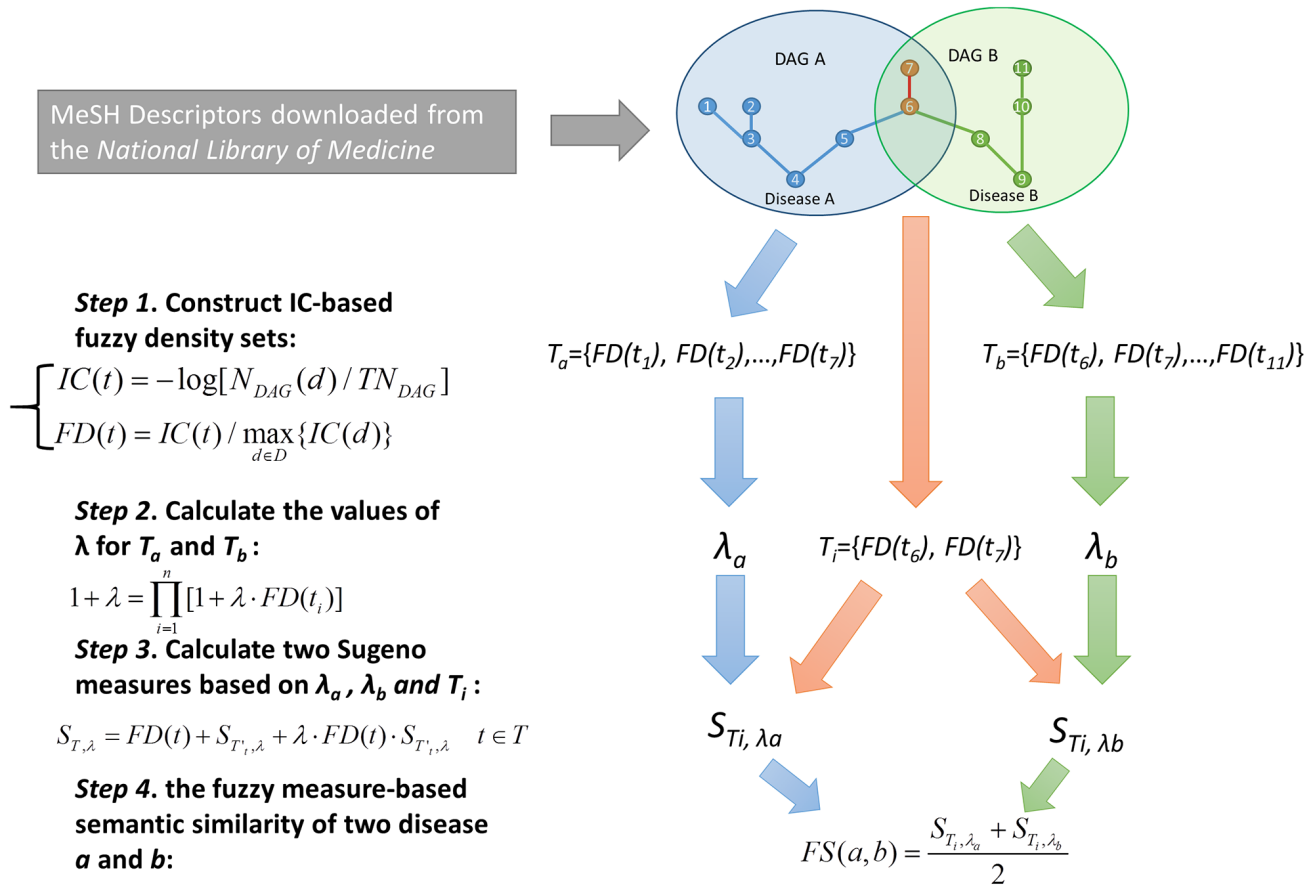
Flowchart of disease semantic similarity calculation in FMLNCSIM based on disease DAGs

**Figure 2:**
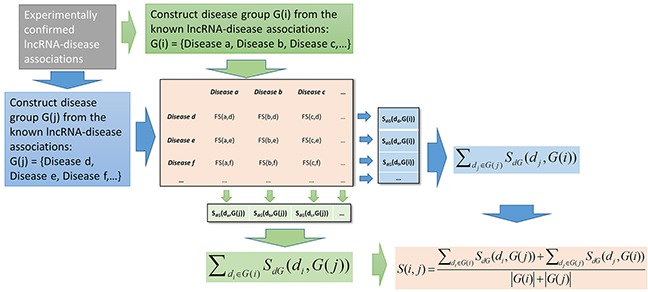
Flowchart of lncRNA functional similarity calculation based on disease semantic similarity

FMLNCSIM is a computational model for calculating the functional similarity of lncRNAs by using the information of known lncRNA-disease associations and diseases DAGs (See Figure 1 and 2). It is mainly based on the assumption that functionally similar lncRNAs tend to be involved in similar disease and vice versa. The performance improvement of FMLNCSIM mainly comes from the combination of the concept of information content and the fuzzy set theory. Information content of disease terms in DAGs helps to retain their specificity and fuzzy measure is expected to lead to a more accurate similarity measurement based on the disease sets. The concept of information content has been adopted by some of previous researches related with gene ontology (GO) terms [59–61]. For example, Yu *et al.* have proposed an R package for semantic similarity among GO terms and gene products by introducing the concept of information content [59]. Fröhlich *et al.* have also used this concept and developed GOSim package for measuring functional similarity of gene products [60].

The similarity measure is one of useful tools for the degree of similarity between objects and is deeply studied in the fields of physical anthropology, numerical taxonomy, ecology, information retrieval, psychology, citation analysis, and automatic classification. Various term-term similarity measures have been proposed for expressing the degree of similarity of sets. For example, the Jaccard and Dice similarity measures, which were proposed in 1901 and 1945, respectively, have been widely used [62, 63]. However, it is shown that these functions have inherent limitations that they are only treated as discrete with loss of information and fail to measure the similarity between the trapezoidal intuitionistic fuzzy numbers (TIFNs) which should be treated as continuous [62, 64]. Information available is sometimes vague, inexact or insufficient and fuzzy set theory proves to be ideally suited for solving these problems [65]. Since the scale of recorded MeSH descriptors associated with a specific disease greatly depend on the research degree on it, this bias in disease DAGs can cause partial, insufficient or redundant information for calculating lncRNA functional similarity and further greatly influence the effectiveness of disease-based computational models.

### Performance evaluation

FMLNCSIM was developed to calculate the functional similarity scores of lncRNAs in LncRNADisease and MNDR databases, which are listed in Supplementary Table S1 and S2, respectively. To evaluate the performance of FMLNCSIM, we combined it with LRLSLDA and validated the effectiveness of the integrated model of FMLNCSIM-LRLSLDA. The performance of FMLNCSIM-LRLSLDA is directly influenced by the computed functional similarity of lncRNAs and therefore can reflect the effectiveness of FMLNCSIM. In the original version of LRLSLDA, Gaussian interaction profile kernel similarity and lncRNA expression similarity are integrated for constructing lncRNA similarity [37]. In this work, we applied a simple average operation to generate a new integrated disease similarity by combining calculated disease semantic similarity and disease Gaussian interaction profile kernel similarity. We further computed a new integrated lncRNA similarity by combining lncRNA functional similarity which was generated by ILNCSIM, lncRNA Gaussian interaction profile, and lncRNA expression similarity. By this means, we built a new model for quantifying the possibilities of potential lncRNA-disease associations, LRLSLDA-FMLNCSIM. It was mainly constructed by two parts – lncRNA functional similarity calculation model based on fuzzy measure and lncRNA-disease associations prediction model based on Laplacian regularized least squares.

In this work, we explored the known lncRNA-disease associations stored in the manually crated diverse ncRNA-disease repository (MNDR) [66] and LncRNADisease [12] database by adopting two validation frameworks of global LOOCV and 5-fold cross validation. Specifically, for the framework of global LOOCV, each known lncRNA-disease association was left out in turn for testing and other samples were used for training, and all the lncRNA-diseases without recorded association evidence were considered as candidate samples. For 5-fold cross validation method, all known lncRNA-disease associations were randomly divided into five disjoint parts, of which four were used for training and the other one was used as testing samples. To visually evaluate the performance results of the proposed model, Receiver-operating characteristics (ROC) curves were drawn. For further evaluation, the value of AUC was also computed by measuring the area under ROC curves based on testing samples. The lncRNA-disease associations with higher ranks than the given threshold in the testing set were considered as successful predictions while predicted ranks lower than threshold lead to unsuccessful predictions. By setting different thresholds, we could obtain corresponding true positive rates (TPR, sensitivity) and false positive rates (FPR 1-specificity). Here, sensitivity was computed based on the percentage of samples which obtained higher ranks than the threshold, and specificity, on the other hand, denotes the percentage of negative samples with lower ranks than the threshold. ROC curves were further created by plotting the TPR against FPR at various threshold settings. The value of area under ROC curve (AUC) was computed for quantifying the performance results. In general, the value of AUC close to 0.5 means purely random performance while AUC close to 1 imply a promising prediction result.

**Figure 3:**
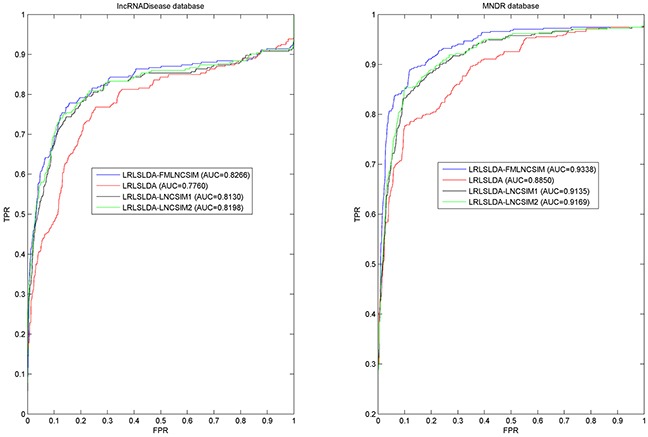
Performance comparisons between FMLNCSIM and three state-of-the-art disease-lncRNA association prediction models (LRLSLDA, LRLSLDA-LNCSIM1 and LRLSLDA-LNCSIM2) in terms of ROC curve and AUC based on global LOOCV There are roughly 58 and 94 testing samples in LncRNADisease and MNDR databases respectively. As a result, FMLNCSIM achieved AUCs of 0.8266 and 0.9338 based on the LncRNADisease and MNDR databases, which significantly outperformed all the previous classical models and effectively demonstrated its reliable predictive ability.

In this work, we compared the performance of LRLSLDA-FMLNCSIM with three previously proposed computational methods (i.e. LRLSLDA [37], LRLSLDA-LNCSIM1 [55] and LRLSLDA-LNCSIM2 [55]). Figure 3 shows the comparison performance in the framework of global LOOCV. It can be observed that LRLSLDA-FMLNCSIM, LRLSLDA, LRLSLDA-LNCSIM1 and LRLSLDA-LNCSIM2 achieve AUCs of 0.8266, 0.7760, 0.8130 and 0.8198 on LncRNADisease dataset, and yielded AUCs of 0.9338, 0.8850, 0.9135 and 0.9169 on the MNDR dataset, respectively. We also adopted 5-fold cross validation method for further evaluation. To minimize the influence of random division, 5-fold cross validation was repeated 100 times and the average and standard deviation of AUCs yielded by the four models were computed. When we explored the LncRNADisease database, LRLSLDA-FMLNCSIM achieved the best performance with AUC of 0.7979+/−0.0098, significantly higher than those yielded by other methods (LRLSLDA: 0.7295+/−0.0089; LRLSLDA-LNCSIM1 0.7761+/−0.01; LRLSLDA-LNCSIM2 0.7872+/−0.0097). For the MNDR dataset, the yielded comparison results also demonstrated FMLNCSIM was superior to the other methods. LRLSLDA-FMLNCSIM achieved AUCs of 0.9237+/−0.0050 while LRLSLDA, LRLSLDA-LNCSIM1 and LRLSLDA-LNCSIM2 yielded poorer performance with AUCs of 0.8687+/−0.0053, 0.9012+/−0.0044 and 0.9050+/−0.0041, respectively. In conclusion, FMLNCS IM has proved to achieve greater effectiveness for calculating lncRNA functional similarity in the validation frameworks of global LOOCV and 5-fold cross validation.

### Case studies

To further evaluate the performance of FMLNCSIM, we here applied LRLSLDA-FMLNCSIM to predict the most possible lncRNAs associated with two important diseases, acute myeloid leukemia and lung cancer, based on the known lncRNA-disease associations in the MNDR dataset. The lncRNA-disease association which obtained top 10 ranks were considered as the most potential candidates and further verified based on another existing databases about lncRNA-disease associations, Lnc2cancer [67], as well as recently published experimental literatures.

Acute myeloid leukemia is one of the high-mortality diseases with long-term overall survival (OS) rates of only 5–16% [68]. The older adults are considered as high-risk populations for acute myeloid leukemia due to the higher frequencies of secondary disease, adverse cytogenetics, comorbid conditions, and poor performance status [69]. An increasing number of novel genetic alteration including gene mutations and changes in gene expression are identified by recent works, which helps to improve the classification and risk stratification of acute myeloid leukemia patients [70]. We here applied LRLSLDA-FMLNCSIM to identify most potential lncRNAs associated with acute myeloid leukemia. As a result, lncRNA UCA1 and HOTAIR in the top 10 candidate list were verified by Lnc2cancer database.

Despite of the advances in clinical and experimental oncology, the prognosis of lung cancer is still unfavorable, with about 1.8 million new cases every year [71]. As one of the markedly leading causes of death worldwide, the 5-year survival rate of lung cancer is still dismal, only around 11% [72, 73]. In addition, lung cancer is usually hard to be diagnosed until advanced stage and therefore prognosis for lung cancer is important for the treatment. The participation of lncRNAs in the development of lung cancer has been intensely researched. Accumulating evidence link dysregulations of some lncRNAs to lung cancers and consider them as the biomarkers for lung cancer therapy. However, the number of detected lncRNAs associated with lung cancer is still limited. In this work, we applied LRLSLDA-FMLNCSIM to prioritize candidate lncRNAs based on known associations in the MNDR database. As a result, four potential lncRNAs with top 10 ranks (BC200, UCA1, HOTAIR, and XIST) were verified by Lnc2cancer database and relevant literatures [74]. Specially, UCA1 was predicted as the third candidate and confirmed by the recent observation that the overexpression of plasma UCA1 promoted the malignant progression of lung cancer [74].

The promising results obtained from global LOOCV, 5-fold cross validation and case studies have demonstrated the reliable performance of LRLSLDA-FMLNCSIM. Therefore, we further prioritize all the candidate lncRNAs for all the diseases recorded in MNDR database by utilizing the known experimentally confirmed lncRNA-disease associations stored in MNDR database and implementing the model of LRLSLDA-FMLNCSIM. The predicted ranks of lncRNAs for each disease were publicly released for further experimental validation (See Supplementary Table S3). The potential lncRNA-disease associations with high ranks are expected to be confirmed by biological experiments and clinical observation in the future.

## DISCUSSION

Measuring lncRNA functional similarity has high value in inferring lncRNA functions as well as searching highly potential lncRNA-disease associations. Since the participation of lncRNAs has been confirmed to influence the development of diseases by increasing clinical observations, it is feasible to measure the functional similarity of lncRNAs based on lncRNA-disease associations. In this work, we proposed a novel computational model for calculating lncRNA functional similarity by combining the information of know lncRNA-disease associations and the disease semantic similarity. To our knowledge, fuzzy measure was first to be introduced for computational models associated with lncRNAs. For further evaluation, FMLNCSIM was integrated with previously proposed LRLSLDA model to quantify lncRNA-disease association probabilities. The reliable results yielded by LRLSLDA-FMLNCSIM in two evaluation frameworks (i.e. global LOOCV and 5-fold cross validation) demonstrated the high effectiveness of FMLNCSIM. Based on the model of FMLNCSIM, lncRNAs with the information of associated diseases could be efficiently searched for their functionally similar ones. The lncRNAs with predicted high ranks could be considered highly potential candidate for further biological experiment verification. Thus, we publicly released the potential lncRNA-disease pairs for all the diseases investigated in this work. We anticipate that there will be more predictions with high ranks confirmed by future biological experiments.

There are some limitations in the model of FMLNCSIM. Firstly, since the fuzzy measure needs to transform disease MeSH DAGs into fuzzy density sets for further computation, the hierarchical structure of DAGs would be failed to be retained in this transformations. Besides, considering the fact that the degrees of researches for different disease are imbalanced, the information bias in DAGs may influence the accuracy of FMLNCSIM. Finally, the proposed version of FMLNCSIM failed to integrated additional data from other types of biological datasets associated with lncRNAs.

## MATERIALS AND METHODS

### LncRNA-disease associations

In the previous work, we have constructed the first publicly available lncRNA-disease association database, LncRNADisease (http://cmbi.bjmu.edu.cn/lncrnadisease) [12], by manually collecting experimentally confirmed lncRNA-disease associations from accumulating experimental reports. We downloaded known lncRNA-disease associations and got rid of those duplicate samples which describe the same lncRNA-disease association based on different experimental evidences. As a result, there are 293 distinct high-quality lncRNA-disease associations, which include 118 lncRNAs and 167 diseases. For further performance evaluation, we downloaded known associations from another lncRNA-disease association database, the Mammalian ncRNA-disease repository (MNDR, http://www.rna-society.org/mndr/) [66], in March, 2015. The duplicate lncRNA-disease associations from different evidences were also removed. As a result, we obtained 471 high-quality experimentally verified samples of 127 diseases and 241 lncRNAs.

### Disease MeSH descriptors

For the measurement of disease similarity, we downloaded MeSH descriptors from the National Library of Medicine (http://www.nlm.nih.gov) [75] to construct disease DAGs. Based on a strict system for disease classification, there are 16 categories included in MeSH descriptors (e.g. Category A: anatomic terms; Category B: organisms; Category C: diseases; Category D: drugs and chemicals). In this work, we downloaded the descriptors of Category C and constructed DAGs to depict the disease association. In the disease DAGs, the nodes represent disease MeSH descriptors and each edge denotes the connection from a more general term (parent node) to a more specific term (child node).

### Fuzzy measure-based disease semantic similarity

Fuzzy measures have recently proved to be useful and superior to additive probability measures for describing expert uncertainty. For example, k-additive fuzzy measure reduces the number of variables for definition by limiting the interaction between its subsets [76, 77]. In this work, Sugeno λ-measures, one of the most widely and successfully used class of fuzzy measures, were introduced to calculate disease semantic similarity based on disease MeSH DAGs [78, 79]. Specifically, the information content (IC) for each disease term were computed based on the corresponding MeSH DAG, and further used as fuzzy density values. The fuzzy measure-based disease similarity was then computed based on the IC-based fuzzy density values. The calculation process for disease semantic similarity mainly consists of four steps (See Figure 1).

Disease terms with higher specificity usually have a larger contribution to measuring disease similarity. In the first step, we introduced the concept of information content which can effectively depict how specific a term is. Specifically, we counted the number of occurrences in the DAGs of the term (say, disease *d*) and then converted it to information content by computing the negative log likelihood:

$$IC(t)=-\log[N_{DAG}(d)/TN_{DAG}]$$ (1)

where *N_DAG_*(d)** denotes the number of DAGs including *d*; *TN_DAG_* denotes the total number of diseases. We further proposed fuzzy density sets aiming at retaining the specificity information of disease group members for further set-set similarity measurements. To achieve this goal, we then defined the fuzzy density by using a normalization operation:

$$FD(t)=IC(t)/\underset{d\in D}{\max}\{IC(d)\}$$ (2)

where *D* denotes the whole disease set included in the dataset. In this way, diseases' DAGs were converted into real sets which were considered as fuzzy sets for further calculation.

In the second step, the values of λ for Sugeno measure were computed for each fuzzy density set. Given a fuzzy density set *T*={*FD(t_1_)*, *FD(t_2_)*, …,*FD(t_n_)*}, the value of λ for T was computed based on the following equation:

$$1+\lambda =\prod_{i=1}^{n}\left[1+\lambda \cdot FD(t_{i})\right]$$ (3)

For each fuzzy density set, λ has a unique value since equation (3) has a unique solution for λ>-1.

In the third step, the Sugeno measures for the intersection of two fuzzy density sets were computed. Given two fuzzy density sets, *T_a_* and *T_b_*, and their intersection *T_i_*, two Sugeno measures for *T_i_* were computed based on the values of *λ* form *T_a_* and *T_b_*. Given a fuzzy density set *T*={*FD(t_1_)*, *FD(t_2_)*,…,*FD(t_n_)*}, we say the subset of *T* which excludes the element *t* as *T'_t_*. Then, the Sugeno measure of *T* was calculated based on *λ* and the Sugeno measure of its subset *T'_t_*, which could be defined as follow:

$$S_{T,\lambda }=FD(t)+S_{T\prime_{t},}{}_{\lambda }+\lambda \cdot FD(t)\cdot S_{T\prime_{t},}{}_{\lambda }\;t\in T$$ (4)

In this recursive way, the Sugeno measure of *T* could be finally computed. Specially, for the fuzzy density set whose size equals one, its Sugeno measure was set to be the value of its only element. Assume that *λ* values of *T_a_* and *T_b_* are computed as *λ_a_* and *λ_b_*. Then, two Sugeno measures, $S_{T_{i},\lambda_{a}}$ and $S_{T_{i},\lambda_{b}}$ would be computed by the same way defined as equation 4. As a result, the value of *S_T, λ_* could be finally obtained in a recursive way.

In the final step, the fuzzy measure-based semantic similarity of two disease terms, *a* and *b*, were calculated based on the average of Sugeno measures:

$$FS(a,b)=\frac{S_{T_{i},}{{}_{\lambda }}_{{}_{a}}+S_{T_{i},}{{}_{\lambda }}_{{}_{b}}}{2}$$ (5)

In this way, the semantic similarity of each disease pair could be computed to constitute the disease similarity matrix *FS*, where the entity in row *i* column *j* represent the semantic similarity between *ith* disease and *jth* disease.

### FMLNCSIM

Based on the fuzzy measure-based disease semantic similarity, FMLNCSIM was then developed to compute the lncRNA functional similarity by using the information of known lncRNA-diseases. Specifically, the lncRNA functional similarity of two lncRNAs was measured by the similarity between their associated disease groups. Given two disease groups, *G(i)* and *G(j)*, which are respectively associated with lncRNA *i* and lncRNA *j*, we calculated their similarity based on a group-based method (See Figure 2). The similarity between one of disease term (say *d_i_*) in *G(i)* and *G(j)* was computed based on a maximum operation and could be defined as follow:

$$S_{dG}(d_{i},G(j))=\underset{d_{j}\in G(j)}{\max}(FS(d_{i},d_{j}))$$ (6)

The functional similarity between lncRNA *i* and lncRNA *j* was then computed based on the set-based similarity between *G(i)* and *G(j)*:

$$S(i,j)=\frac{\sum_{d_{i}\in G(i)}S_{dG}(d_{i},G(j))+\sum_{dj\in G(j)}S_{dG}(d_{j,}G(i))}{\left|G(i)\right|+\left|G(j)\right|}$$ (7)

where *|G(i)|* and *|G(j)|* are the numbers of diseases in *G(i)* and *G(j)*, respectively.

### Webserver

In order to provide convenience for applying our proposed model, we built a web server which implements the function of the proposed FMLNCSIM model. It is available at http://219.219.60.245/. This web server mainly carries out four functions. The function 1 and 2 enable visitors obtain functional similarities calculated by FMLNCSIM model based on two lncRNA-disease association databases (i.e. LncRNADisease and MNDR). The function 3 and 4 provide functional similarity calculation for new lncRNAs as long as users provided its associated diseases. When visitors provide a specific lncRNA with its associated diseases, function 3 and 4 could calculate the functional similarities between this query lncRNA and all lncRNAs in LncRNADisease and MNDR databases, and then list the results on the webpage.

## SUPPLEMENTARY TABLES








