# Supplementary material for: FMLNCSIM: fuzzy measure-based lncRNA functional similarity calculation model
Source: Oncotarget. 2016 Jun 14;7(29):45948–58. doi: 10.18632/oncotarget.10008 (PMC5216773; doi:10.18632/oncotarget.10008)
Supplement: Supplementary file 1 [file oncotarget-07-45948-s001.pdf]

FMLNCSIM: fuzzy measure-based lncRNA functional similarity calculation model

SUPPLEMENTARY TABLES

Supplementary Table S1: LncRNA functional similarity scores calculated by FMLNCSIM based on lncRNADisease dataset.

See Supplementary File 1

Supplementary Table S2: LncRNA functional similarity scores calculated by FMLNCSIM based on MNDR dataset.

See Supplementary File 2

Supplementary Table S3: We further applied LRLSLDA-FMLNCSIM to prioritize all the candidate lncRNA-disease pairs based on all the lncRNA-disease associations recorded in MNDR database as training samples. Prediction results were publicly released for further research and experimental validation.

See Supplementary File 3
